# FGF10 Signaling differences between type I pleuropulmonary blastoma and congenital cystic adenomatoid malformation

**DOI:** 10.1186/1750-1172-8-130

**Published:** 2013-09-03

**Authors:** Guillaume Lezmi, Virginie Verkarre, Naziha Khen-Dunlop, Shamila Vibhushan, Alice Hadchouel, Caroline Rambaud, Marie-Christine Copin, Jean-Luc Rittie, Alexandra Benachi, Jean-Christophe Fournet, Christophe Delacourt

**Affiliations:** 1 AP-HP, Hôpital Necker-Enfants Malades, service de Pneumologie Pédiatrique, Centre de Référence pour les Maladies Respiratoires Rares de l’Enfant, Paris, France; 2 INSERM, U955, IMRB, Equipe 04, Créteil, France; 3 AP-HP, Hôpital Necker-Enfants Malades, service d’Anatomie et de Cytologie Pathologiques, Paris, France; 4 AP-HP, Hôpital Necker-Enfants Malades, service de Chirurgie Viscérale Pédiatrique, Paris, France; 5 AP-HP, Hôpital Raymond Poincaré, service d’Anatomie Pathologique et Médecine Légale, Garches, France; 6 Institute de Pathologie, Centre Hospitalier Régional Universitaire, Lille, France; 7 CHU Felix Guyon, Saint-Denis, La Réunion, France; 8 Université Paris-Sud, Bicêtre, France; 9 Université Paris-Descartes, Paris, France

**Keywords:** Pleuropulmonary blastoma, Congenital cystic adenomatoid malformation, Lung malformation, Cystic lung disease, Fibroblast growth factor 10, Children

## Abstract

**Background:**

Type I pleuropulmonary blastoma (PPB) and congenital cystic adenomatoid malformation of the lung (CCAM) are cystic lung diseases of childhood. Their clinical and radiological presentations are often similar, and pathologic discrimination remains difficult in many cases. As a consequence, type I PPB and CCAM are frequently confused, leading to delayed adequate management for type I PPB. Recent studies have suggested a role for fibroblast growth factor (FGF) 10 signal pathway in CCAM pathogenesis. The objective of our study was to determine whether FGF10 signaling differs between CCAM and type I PPB.

**Methods:**

Immunohistochemical studies were performed for expression of FGF10, its receptor FGFR2b, and its inhibitor sonic hedgehog (SHH) in focal type I PPB (n=6), CCAM type I (n=7), CCAM type II (n=7), and control lungs (n=5).

**Results:**

FGF10, FGFR2b, and SHH expressions differed markedly between type I PPB and both types of CCAM. Type I and type II CCAM cystic walls expressed FGF10, FGFR2b, and SHH, whereas staining was absent or poor in type I PBB cystic walls*.* Expression of FGF10, FGFR2b, and SHH did not differ between CCAM cystic walls and control airway walls.

**Conclusions:**

These findings show that immunohistochemistry with FGF10, FGFR2b, or SHH could be useful in differentiating CCAM from type I PPB, when a child presents with a focal cystic lung lesion. The absence of strong expression of FGF10, FGFR2b, and/or SHH makes the diagnosis of CCAM very doubtful.

## Introduction

Type I pleuropulmonary blastoma (PPB) and congenital cystic adenomatoid malformation of the lung (CCAM) are cystic lung diseases occurring in neonates or in young children [1]. Distinguishing between them remains a challenge for pathologists, and type I PPB is currently confused with CCAM [2-4]. PPB is a rare and aggressive embryonic tumor of the lung [5], which is believed to arise from primitive lung mesenchyme [6]. PPB is classified into 3 types: purely cystic (type I), cystic and solid (type II), and predominately solid (type III) [7]. These 3 types represent different stages of one malignant process in which type I PPB is the earliest manifestation. Without complete resection, low-grade cystic PPB (type I) is likely to progress over 2–4 years to a high grade solid disease (type III) [6]. Delayed recognition or failure to resect type I PPB significantly worsens prognosis. Overall survival rate in PPB ranges from 80-85% for type I to 45-50% for type III [8,9].

CCAM is the most frequent congenital cystic lung lesion [10]. Classification of CCAM is based on pathologic features, type I and type II CCAM being the most frequent forms [11,12]. Type I CCAM is composed of single or multiple large cysts, while type II CCAM is characterized by multiple small cysts. After resection CCAM does not recur, and malignant transformation is an unresolved question, but seems to be an unusual event [10].

Distinguishing between type I PPB, and type I or type II CCAM is crucial since their management and prognosis are different. As these 2 entities share similar clinical and radiological features, pathologic examination is the only means to establish the final diagnosis [6,8,9]. However, the pathologic diagnosis is itself often difficult, because of the absence of pathognomonic markers that differentiate between these two cystic diseases. Indeed, CCAM and type I PPB have many overlapping pathologic features, such as multiloculated cysts lined with flattened or cuboidal epithelial cells, and imperceptible transition between normal and pathologic areas [2,4,6,8]. Histologic features of type I PPB are themselves heterogeneous, with wide variations regarding the septal cellularity density, the presence of differentiated skeletal muscle or cartilage, and the capillary vessel density [8]. None of these features are specific, increased cell proliferation [13] and striated muscle cells proliferation [14] being described in CCAM. Mainly, foci of dense subepithelial or septal spindle cell proliferation, which are highly suggestive of type I PPB [5,8,15], can be extremely localized [9], making an extensive sampling of cyst walls critical for diagnosis [15].

The pathogenesis of CCAM is uncertain. Altered expression of factors controlling normal lung development has been identified in CCAM [12,15-18]. In particular, the potential role of fibroblast growth factor (FGF) 10 in CCAM pathogenesis was recently highlighted [18]. Induction of localized lung FGF10 overexpression in fetal rats resulted in localized macrocystic or microcystic lung malformations, depending on the developmental stage and the site of overexpression [18]. FGF10 is a mesenchymal growth factor which may act on the epithelium through its receptor FGFR2b to control pulmonary morphogenesis [19]. It is known to play a key role in branching morphogenesis [20]. During lung development, mesenchymal FGF10 expression is inhibited by Sonic Hedgehog (SHH), a diffusible factor secreted by epithelial cells [19]. In this study, we performed immunohistochemical staining to compare the expression of FGF10, FGFR2b, and SHH in type I and type II CCAM, and in type I PPB. The objective was to determine if they were differentially expressed.

## Materials and methods

### Patients

This study was approved by an Ethics Committee (Comité de Protection des Personnes, Ile-de-France VII). First part of the study was conducted from tissue specimens already collected. Tissues of patients with type I PPB (n=3), and with CCAM (type I, n=5; type II, n=5) were part of the archives of the department of pathology of the Necker-Enfants Malades hospital. Three supplementary specimens of patients with type I PPB were provided by the departments of pathology of the Centre Hospitalier Regional Universitaire of Lille (n=1), and by the Centre Hospitalier Universitaire Felix Guyon of La Reunion (n=2), respectively. The results obtained from CCAM tissues were then prospectively confirmed on four new cases with CCAM (2 type I and 2 type II), who were operated at Necker-Enfants Malades Hospital. All patients (n=20) presented with focal cystic lung lesions, and tissues were obtained after surgical resection. All CCAM cases were diagnosed prenatally, contrasting with none of the PPB cases. Cases of CCAM evaluated in this study can be considered as representative, because our center does not select surgical indications, but proposes a systematic removal of all congenital lung malformations with cystic appearance, because of an unpredictable risk of malignancy. Controls were also performed with normal lung sections (n=5), provided by the department of pathology of the Raymond Poincare Hospital, Garches, France. These sections were collected at autopsies of age-matched children who died of a non-pulmonary cause (sudden infant death syndrome). All tissues were obtained from 2006 to 2013. General characteristics of patients are summarized in the Table 1.

**Table 1:** General characteristics of cases

**N° Patient**	**Final diagnosis**	**Age at tissue collection (months)**	**Localization**
1	CCAM type I	0.1	Right upper lobe
2	CCAM type I	0.2	Right lobe
3	CCAM type I	0.8	Right middle lobe
4	CCAM type I	21.9	Right lobe
5	CCAM type I	42.5	Right upper lobe
6	CCAM type I	6.7	Left inferior lobe
7	CCAM type I	13.6	Left upper lobe
8	CCAM type II	1.8	Left lobe
9	CCAM type II	6.8	Left upper lobe
10	CCAM type II	8.9	Left upper lobe
11	CCAM type II	14.2	Left lobe
12	CCAM type II	15.6	Unknown
13	CCAM type II	1.7	Left inferior lobe
14	CCAM type II	8.8	Right middle lobe
15	PPB type I	31.3	Fowler lobe
16	PPB type I	28.1	Left lobe
17	PPB type I	24.7	Left lobe
18	PPB type I	6.8	Left upper lobe
19	PPB type I	35.6	Left inferior lobe
20	PPB type I	14.8	Unknown
21	Sudden infant death	0.7	
22	Sudden infant death	2.5	
23	Sudden infant death	4	
24	Sudden infant death	7.5	
25	Sudden infant death	10.5	

### Diagnostic criteria

CCAM and type I PPB were diagnosed after microscopic examination of surgically excised cystic lesions. CCAM was classified according to the Stocker classification into macrocystic type I when cyst size was > 2 cm, and microcystic type II when cyst size was < 2 cm [12]. In the text, the term “CCAM” will refer to both type I and type II CCAM. If data are specific to a given type, this one will be mentioned. Diagnosis of type I PPB was made if one or more foci of dense subepithelial or septal primitive spindle cells were present. The initial diagnosis of CCAM and PPB was confirmed before any staining by the two independent pathologists participating to this study (VK and JCF).

### Immunohistochemistry

After resection, tissues were paraffin-embedded, and 5 μm sections were cut for immunohistochemistry. Sections were deparaffinized, rehydrated, and antigens were unmasked by boiling for 30 minutes in 10 mM citric acid buffer pH 6, FW: 210,1 (Sigma, Saint-Louis, USA). After 25-minute cooling at room temperature (RT), sections were rinsed twice, 5 minutes each, in phosphate-buffered saline (PBS), pH 7.4 (Life technologies, Carlsbad, USA), and blocked with 2.5% normal horse serum (RTU Vectastain, Vector Laboratories, Burlingame, USA) for 15 minutes at RT. Sections were then incubated overnight at 4°C with the primary antibodies (FGF10, mab345, R&D Systems, 1:1250; FGFR2b, ab58201, Abcam, 1:400; SHH, ab53281, Abcam, 1:100), and controls (control FGF10, mab004, R&D Systems, 1:1250; control FGFR2b, 550878, BD Pharmingen, 1:100; control SHH, R&D Systems, Ab105/c, 1:1000) , with 1.5% normal horse serum (RTU Vectastain, Vector Laboratories, Burlingame, USA) in PBS. After overnight incubation, sections were washed 3 times in PBS, 2 minutes each, and endogenous peroxidase was quenched with 3% H202 for 20 minutes at RT. Sections were rinsed 3 times in PBS, 2 minutes each, and then incubated with biotinylated universal secondary antibody (RTU Vectastain, Vector Laboratories) for 10 minutes at RT. After 1 wash in PBS, sections were incubated with the streptavidin-peroxidase complex from the RTU Vectastain (Vector Laboratories, Burlingame, USA) for 5 minutes at RT, and rinsed once for 5 minutes in PBS. Staining was visualized with diaminobenzidine (Vector novaRED, Vector Laboratories, Burlingame, USA) for 10 minutes, and then rinsed with distilled water for 5 minutes. Sections were counterstained with hematoxylin for 20 seconds (Hematoxylin QS, Vector Laboratories, Burlingame, USA). Immunostainings with Desmin (M0760, Dako, 1:200) and Ki-67 (M7240, Dako, 1:100) were also performed routinely in each type I PPB, in 7 CCAM, and all controls.

### Analysis

The analysis of staining was blinded, and was performed independently by two of the authors (GL and SV). The quantification was performed per area of tissue. Because of their diffuse expression within the interstitial tissue, FGF10 and SHH staining were analyzed in a descriptive way, and no reliable quantification could be performed. By contrast, the localized expression of FGFR2 within the epithelium allowed a quantitative analysis. For each patient with type I PPB, CCAM, and normal lung, we photographed, at magnification 20, five randomized zones of epithelium stained with FGFR2b and five randomized zones stained with control antibody. Expression of FGFR2b/control in epithelial cells was quantified in gray intensity by epithelium area using image J software. Mean values for control antibody were determined and served as a reference. They were subtracted from mean values of FGFR2b, and the resulting values were compared using the nonparametric Mann–Whitney test, and a p value < 0.05 was considered significant.

## Results

### FGF10 expression

**Figure 1:**
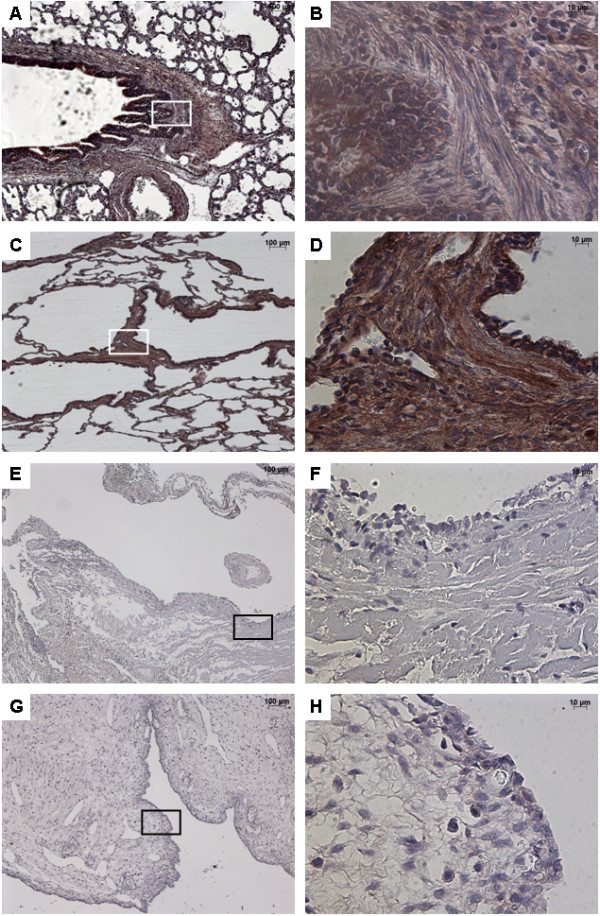
**Immunostaining with FGF10.** From left to right, original magnification x 5 **(A**, **C**, **E**, **G)**, x 40 **(B**, **D**, **F**, **H)**. The x40 magnified area is identified by a rectangle. Counterstain: hematoxilin. **A**-**B**: Control lung; strong FGF10 expression in both the epithelium and the underlying mesenchyme of normal bronchi. **C**-**D**: CCAM; the expression of FGF10 is strong and continue within the epithelium lining cysts, and within the underlying mesenchyme. **E**-**F**: multiloculated cysts in type I PPB, **G**-**H**: large cyst in a type I PPB; nearly absence of FGF10 expression in type I PPB.

FGF10 was strongly expressed within the interstitial tissue and the epithelium of airways and alveoli, both in control lungs and in CCAM (Figure 1). No difference was observed between controls and CCAM. In CCAM, all cysts were strongly stained, and no difference was found between type I and type II lesions.

In contrast, type I PPB was clearly differentiated from control lungs or CCAM (Figure 1). FGF10 expression was weak or absent in type I PPB, and was only found in the interstitial tissue.

### FGFR2b expression

**Figure 2:**
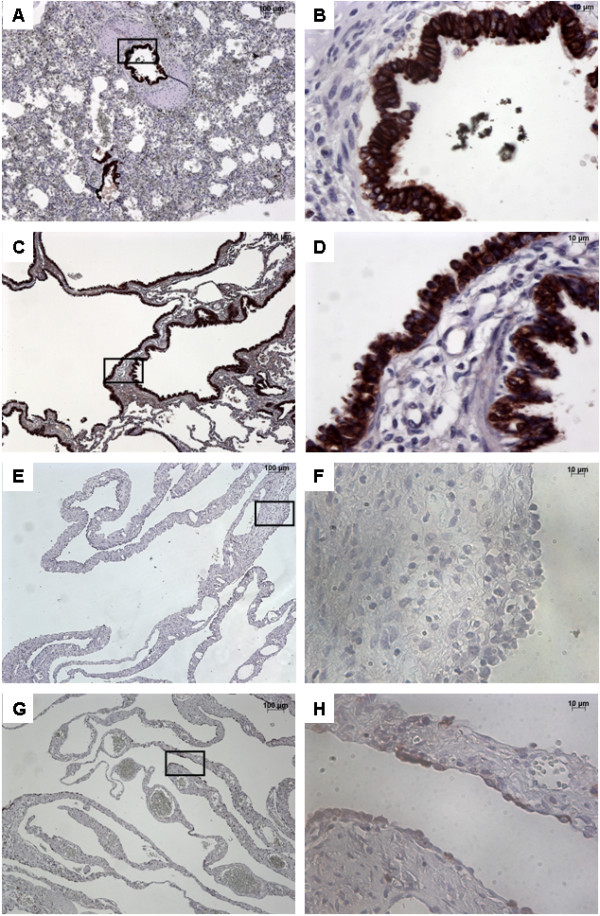
**Immunostaining with FGFR2b.** From left to right, original magnification x 5 **(A**, **C**, **E**, **G)**, x 40 **(B**, **D**, **F**, **H)**. The x 40 magnified area is identified by a rectangle. Counterstain: hematoxilin. **A**-**B**: Control lung; strong FGFR2b expression in epithelial cells of normal bronchi. **C**-**D**: CCAM; strong and continuous FGFR2b expression in the epithelium lining the cysts. **E**-**H**: Multiloculated cysts in type I PPB: No staining **(E**-**F)** or focal staining **(G**-**H)** for FGFR2b.

**Figure 3:**
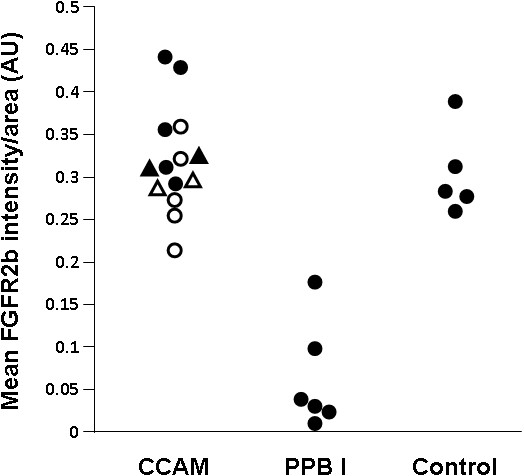
**FGFR2b intensity by area (mm**^**2**^**), in arbitrary units.** Each point represents the mean of 5 measurements of intensity per randomized area. For CCAM: closed symbols = type I CCAM ; open symbols = type II CCAM; circles = retrospective cases; triangles = prospective cases.

In normal lung, FGFR2b was strongly expressed within the bronchial and bronchiolar epithelium, without any disruptions (Figure 2). FGFR2 expression was also observed in alveolar type II epithelial cells (not shown). In CCAM, FGFR2b was strongly expressed in epithelial cells lining the cysts, whatever the size of the cyst. When considering the expression per unit epithelial surface, there were no differences in FGFR2b expression between normal lungs and type I or type II CCAM (Figure 3). FGFR2b expression differed significantly between type I PPB and CCAM. Cysts were not or poorly stained in type I PPB. FGFR2b epithelial expression was absent from most parts of the lesion, and was only present focally weakly in a few small cystic areas. In 2 patients (nos. 17–18), a mild and discontinuous FGFR2b expression was present in the epithelium lining cysts, with large parts of the epithelium without FGFR2b staining (Figure 2D).

After quantification, the intensity of FGFR2b expression was significantly higher in CCAM, in comparison with type I PPB (p < 0.002, Figure 3).

### SHH expression

**Figure 4:**
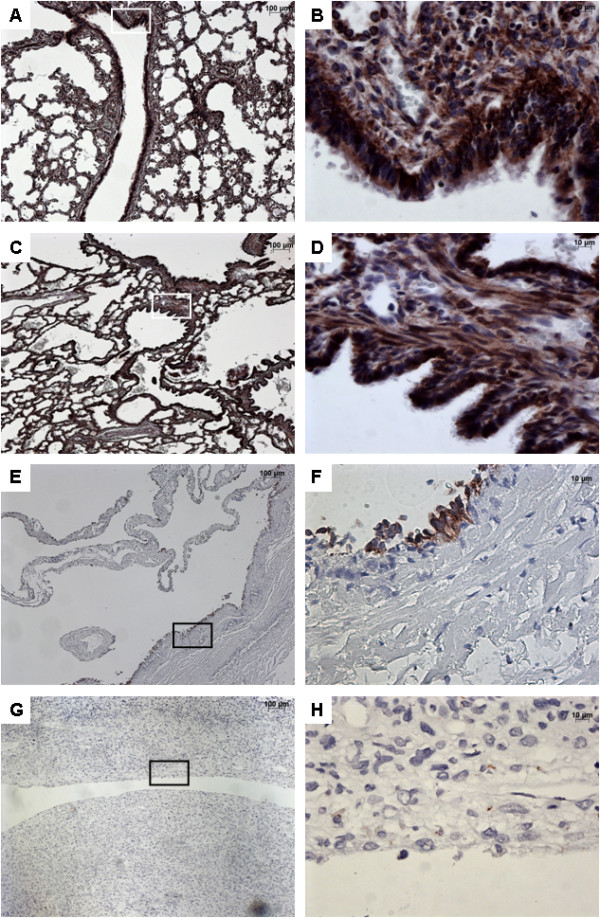
**Immunohistochemistry with SHH.** From left to right, original magnification x 5 **(A**, **C**, **E**, **G)**, x 40 **(B**, **D**, **F**, **H)**. The x 40 magnified area is identified by a rectangle. Counterstain: hematoxilin. **A**-**B**: Control lung; Strong expression of SHH in both the epithelium and the underlying mesenchyme of normal bronchi. **C**-**D**: CCAM. The expression of SHH is strong and continue within the epithelium lining cysts, and within the mesenchyme. **E**-**G**: type I PPB: No SHH staining within the mesenchyme; Focal staining **(E-F)** or no staining **(G-H)** in the epithelium lining the cysts.

In normal lungs, SHH was strongly expressed in both the bronchial and bronchiolar epithelium, and within the underlying mesenchyme (Figure 4). A strong expression of SHH was also present in CCAM, in both the epithelium and the mesenchyme. The expression of SHH was continue within the epithelium lining cysts. Conversely, SHH was virtually not expressed in type I PPB. Its expression was restricted to the epithelium, and was weak and discontinuous.

### Desmin and Ki-67 expressions

**Figure 5:**
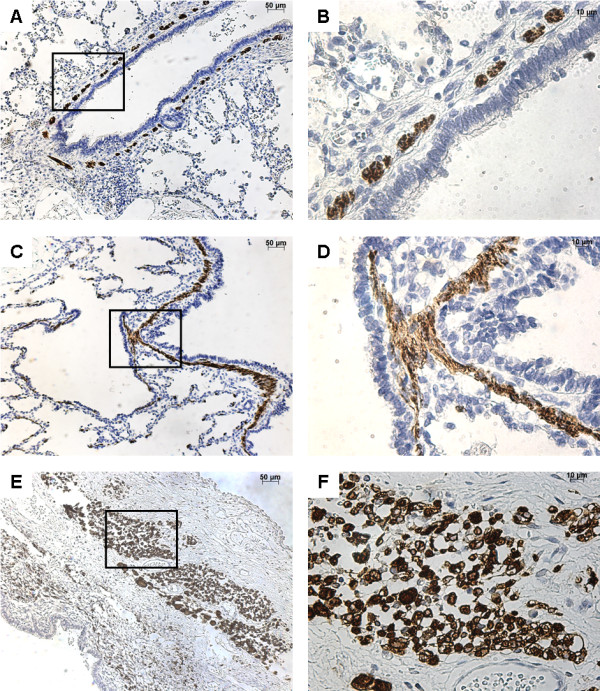
**Immunohistochemistry with desmin.** From left to right, original magnification x 10 **(A**, **C**, **E)**, x 40 **(B**, **D**, **F)**. The x 40 magnified area is identified by a rectangle. Counterstain: hematoxilin. **A**-**B**: Control lung; expression of desmin in smooth muscle cells surrounding normal bronchi. **C**-**D**: CCAM; expression of desmin in smooth muscle cells underlying the sub-basement membrane of the epithelium ling cyst. **E**-**F**: type I PPB; desmin expression in mesenchymal cells of the cystic wall.

To better characterize analyzed tissues, desmin and Ki-67 expression were analyzed. In normal lungs, desmin was expressed in smooth muscle cells surrounding the bronchi and bronchioles (Figure 5). In CCAM, desmin was also expressed in fusiform smooth muscle cells under the sub-epithelial basement membrane. In type I PPB, desmin was expressed in small undifferentiated mesenchymal cells within the cystic walls.

**Figure 6:**
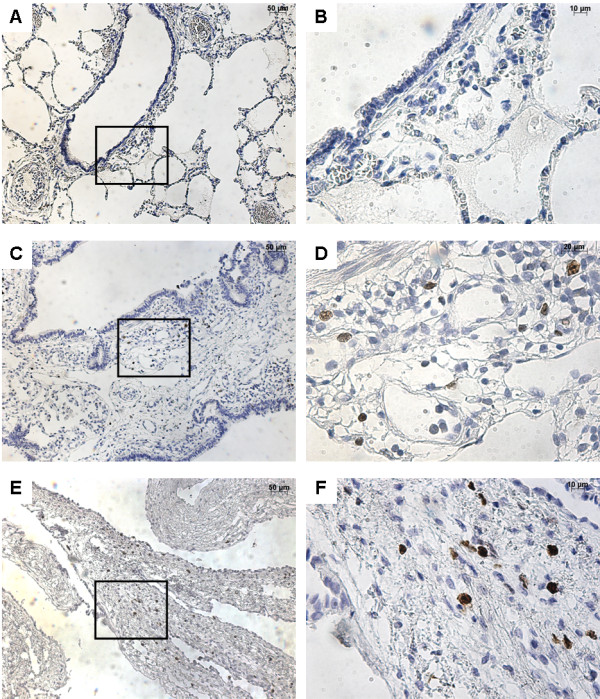
**Immunohistochemistry with Ki-67.** From left to right, original magnification x 10 **(A**, **C**, **E)**, x 40 **(B**, **D**, **F)**. The x 40 magnified area is identified by a rectangle. Counterstain: hematoxilin. **A**-**B**: Control lung; absence of Ki-67 expression. **C**-**D**: CCAM; Ki-67 is expressed in scattered mesenchymal cells. **E**-**F**: type I PPB; Ki-67 expression in mesenchymal cells within the cystic wall.

Ki-67 expression was absent in normal lungs (Figure 6). Ki-67 expression was present in both CCAM and type I PPB cystic walls. It was localized in scattered mesenchymal cells in CCAM, and was present in mesenchymal cells of the cystic walls in type I PPB.

## Discussion

Because of many common histological aspects, type I PPB is frequently mistaken for CCAM, leading to delayed appropriate treatment for PPB. In addition, there are contradictory statements concerning the origin of these two lesions. Some support a common origin, making CCAM able to transform into PPB [21,22]. In contrast, others are in favor of two separate lesions, without natural evolution between them [6]. As the FGF10 signaling pathway was previously suggested to be important in the occurrence of CCAM [18], the objective of our study was to determine whether FGF10, FGFR2b, and SHH expressions differed between CCAM and type I PPB. Our results show that FGF10, FGFR2b, and SHH are strongly expressed in CCAM, but not in type I PPB*.* These findings suggest that use of immunohistochemistry with these markers could improve the recognition of type I PPB. The absence of a strong continuous expression of FGF10, FGFR2b, and SHH in a focal cystic lung lesion renders the diagnosis of CCAM very doubtful. Moreover, our results suggest that different molecular pathways are involved in the development of these two lesions, and are not in favor of their common origin.

Preoperatively, congenital cystic lesions are most commonly anticipated as being CCAM, and type I PPB is rarely suspected [8]. Some clinical features associated with cystic lung lesions can be helpful to suspect a type I PPB [6,8]. Risk factors for PPB include pneumothorax at presentation, bilateral or multifocal lung cysts, familial lung cysts, and associated conditions in patient or family such as renal cystic disease, small bowel polyps, and additional childhood cancer [6]. However, these clinical features lack specificity and are often misinterpreted. In their retrospective analysis of 51 patients with type I PPB, Hill et al. found that type I PPB was never suspected preoperatively despite the high rate of pneumothorax at presentation, and/or multiple lesions in the lung [8].

CCAM and type I PPB share many microscopic features. As in type I PPB, CCAM is often multicystic [12,23]. In both pathologies, cysts are lined by a benign-appearing epithelium. There is no specific aspect of the epithelium that differentiates type I PPB from CCAM. Flattened, cuboidal, columnar, or ciliated type epithelia are all observable in both lesions [8,12]. The most specific microscopic feature of type I PPB is the presence of primitive malignant spindle cells within the septa. These mesenchymal cells are absent in CCAM. Their recognition is crucial and depends on the experience of the examiner since these cells may be extremely localized in small mesenchymal foci, or present only in small amounts. An extensive sampling of the cyst walls remains critical for detailed examination because these cells may only be present in a few sections. The absence of a total resection of a cystic lesion may therefore delay type I PPB diagnosis, and worsens the child’s prognosis.

Cell characterization analyses have been performed in PPB. Markers that have been tested include desmin, myogenin, muscle-specific actin, cytokeratin, thyroid transcription factor 1, and S100 protein [2,5,24]. These studies highlighted the presence of mesenchymal components in type I PPB, but contributed poorly to type I PPB diagnosis [5,8,24]. In particular, rhabdomyomatous dysplastic changes have been also described in CCAM [14,25]. Our study confirmed that desmin expression was present in CCAM. Similarly, we confirmed that increased cell proliferation was not specific of malignant lesions, but was also present in CCAM [26]. In contrast, we show that immunohistochemistry with FGF10, FGFR2b, and SHH could be useful in discriminating between type I PPB and CCAM. The absence of a strong expression of FGF10, FGFR2b, and SHH in a cystic lung lesion makes the diagnosis of CCAM very doubtful, and should prompt the examiner to search for foci of abnormal spindle cells and to ensure the surgical resection is complete. Routine evaluation of FGF10 signaling in focal cystic lung lesions of childhood could therefore markedly reduce the risk of confusion between CCAM and type I PPB, and increase the recognition of type I PPB.

The results of our study support the assumption that type I PPB and CCAM are two distinct diseases. FGF10 signaling is known to play a key role in normal branching morphogenesis [20]. FGF10, FGFR2b, and SHH are normally expressed in the fetal lung mesenchyme and airway epithelium, respectively [18]. In the present study, we demonstrate a persistent expression of FGF10, FGFR2b, and SHH in the normal postnatal lung (airways and alveoli). Postnatal FGF10 signaling pathway have been poorly previously described in postnatal lungs. In rodents, FGF10 mRNA expression was demonstrated to be persistent in adult rats lungs [27]*.* Similarly, FGFR2 mRNA expression was demonstrated in adult mice lungs [28]*.* In humans, Jancelewicz et al. found a low level of FGF10 mRNA expression in infant lungs [29], whereas Hughes found that FGFR2b was strongly expressed in respiratory epithelium of adult lungs [30]. So there is concordance between animal and human data to confirm the persistence of lung expression of FGF10 and FGFR2b through adulthood. As a result, the slight age difference between our PPB and CCAM cases cannot explain the observed differences in FGF10 signaling. The exact role for this pathway in the postnatal lung remains unknown. Since FGR2b expression is predominant in the airway epithelium, the FGF10 signaling pathway may be involved in airway homeostasis. In this connection, it was recently demonstrated that genetic variants affecting the FGF10 signaling pathway were important determinants of adult lung function that may ultimately contribute to chronic obstructive pulmonary disease [31]. Among other FGFs, FGF7 also binds specifically to FGFR2b [32]. Despite binding to the same receptor isoform, these two growth factors do not share the same role in normal lung development. FGF-10 null mice have striking abnormalities, including total absence of lung development below the trachea [33], whereas FGF7 null mice had histologically normal lung development and survival [34]. In our study, the parallel demonstration of major differences in expression of SHH, which is a powerful negative regulator of FGF10 in branching morphogenesis, strengthen our conclusions on differences in FGF10 signaling between CCAM and PPB. Moreover, because FGF10, FGFR2b, and SHH expressions in cystic walls of CCAM do not differ from expression in control airway walls, this strongly suggests that CCAM results from an abnormal enlargement of initially normal airways. Such absence of difference in FGF10 signaling between control postnatal lungs and CCAM lungs was previously demonstrated at the mRNA level [29]. By contrast, the absence of FGF10, FGFR2b, or SHH in cystic walls of PPB suggests that this lesion does not derive from normal airways. These findings may suggest that CCAM and type I PPB are phenotypically different from the early stage of their origin, and that PPB is not a malignant transformation of CCAM. We can, however, not completely exclude that the absence of FGF10, FGFR2b, or SHH does not correspond to a cell dedifferentiation following malignant transformation. This hypothesis seems, however, very unlikely for two main reasons. First, PPB is believed to arise from primitive lung mesenchyme, suggesting that the observed epithelial phenotype is the original one. Second, in the case of dedifferentiation, we would expect foci of normal and dedifferentiated cystic walls. This was not observed in our PPB cases, although some epithelial cells lining PPB cysts in two cases had identifiable FGFR2b or SHH expression. The hypothesis of a distinct origin for CCAM and PPB is consistent with previous cytogenetic studies. The comparison of the karyotype between 11 CCAM cases with 2 PPB cases demonstrated clonal abnormalities in both the PPB cases, but in none of the CCAM cases [35]. Similarly, CGH analysis of 5 PPB cases (1 type I, 2 type II, 2 type III) evidenced gain or loss of chromosomal materials in all cases [36]. In CCAM, chromosomal aberrations may occasionally be detected, in cases associated with foci of atypical goblet cell hyperplasia, adenomatous hyperplasia or adenocarcinoma [37]*.*

Our study has some limitations. Because of the rarity of PPB, the number of evaluated patients remains low. Although the constancy of FGF10 signaling in CCAM is clearly demonstrated, the difference we found between type I PPB and CCAM should be further prospectively confirmed.

In conclusion, our study shows that the absence of strong expression of FGF10 signaling in surgically excised cystic lung lesions from children makes the diagnosis of CCAM very doubtful. Evaluation of FGF10, FGFR2b, and/or SHH expression in these lesions may therefore reduce the risk of confusion between type I PPB and CCAM.

## Abbreviations

CCAM: Congenital cystic adenomatoid malformation of the lung; FGF: Fibroblast growth factor; FGFR: Fibroblast growth factor receptor; PBS: Phosphate-buffered saline; PPB: Pleuropulmonary blastoma; SHH: Sonic hedgehog.

## Competing interests

The authors declare that they have no competing interests.

## Authors’ contributions

GL, VV, AB, JCF, CD contributed to conception and design, and to all steps of the study. NKD, SV, AH, CR, MCC, and JLR contributed to acquisition of data, and to interpretation of data. GL, VV, NKD, JCF, and CD have been involved in drafting the manuscript and revising it critically. All authors have given final approval of the version to be published.
